# Autonomously folded α-helical lockers promote RNAi*

**DOI:** 10.1038/srep35012

**Published:** 2016-10-10

**Authors:** Christian P. E. Guyader, Baptiste Lamarre, Emiliana De Santis, James E. Noble, Nigel K. Slater, Maxim G. Ryadnov

**Affiliations:** 1 Department of Chemical Engineering and Biotechnology, University of Cambridge, Cambridge, CB2 3RA, UK; 2 National Physical Laboratory, Teddington, Middlesex, TW11 0WL, UK

## Abstract

RNAi is an indispensable research tool with a substantial therapeutic potential. However, the complete transition of the approach to an applied capability remains hampered due to poorly understood relationships between siRNA delivery and gene suppression. Here we propose that interfacial tertiary contacts between α-helices can regulate siRNA cytoplasmic delivery and RNAi. We introduce a rationale of helical amphipathic lockers that differentiates autonomously folded helices, which promote gene silencing, from helices folded with siRNA, which do not. Each of the helical designs can deliver siRNA into cells via energy-dependent endocytosis, while only autonomously folded helices with pre-locked hydrophobic interfaces were able to promote statistically appreciable gene silencing. We propose that it is the amphipathic locking of interfacing helices prior to binding to siRNA that enables RNAi. The rationale offers structurally balanced amphipathic scaffolds to advance the exploitation of functional RNAi.

Interfacial tertiary contacts between α-helices underpin intermolecular interactions that regulate or inhibit sub-cellular processes^1^. Examples include helical bundles supporting the docking of viruses to host cells^2^, network-like scaffolds that maintain cytoskeletal structure^3^ and transmembrane coiled-coil clasps mediating cell signalling^4^. Interfacing helices are amphipathic, with non-polar interactions optimised for hydrophobic cores. Therefore, amphipathic helices are said to have two faces – a hydrophobic face, which is buried in an inter-helix core, and a polar face, which provides a hydration shell to the formed oligomer^5^. Such a basic rationale is being exploited for therapy: competitive helices can be designed to disrupt helical interfaces regulating HIV fusion^6^ or as folding antagonists outcompeting membrane-mediated host defence programs^7^. The folding of helical oligomers is context-dependent and requires specific subcellular substrates. This is in contrast to context-independent helix oligomerisation that is increasingly used for designing functional nanostructures^5^,^8^. A key distinction here is that folding is independent of a substrate. Viruses exemplify this strategy. Viral particles fold autonomously, be it with or without nucleic-acid cargo. The latter appears important as viral proteins do not co-fold with their cargo, but use terminal poly-cation domains to bind to it^9^. Such domains lack hydrophobic interactions and do not form amphipathic structures whose hydrophobic faces may otherwise lock the bound nucleic acid (NA) inhibiting its intracellular release. However, mimicking this non-amphipathic strategy still requires a co-folding ability. In the absence of reliable strategies for functional capsid designs, a common approach is the co-complexation of cationic peptides, e.g. oligoarginines, with hydrophobic agents, e.g. liposomes, which allows for the delivery of NA-based agents, but not necessarily gene expression^10^.

Surprisingly, little is known about the role of helical pre-folding in promoting genetic processes. The question remains as to whether α-helices folded prior to NA-binding can mediate the delivery of nucleic acids or their analogues and how this property compares to helix formation induced by co-folding with NAs. Here we set out to address this question by modulating the amphipathic locking of polypeptide α-helices prior to and upon binding NAs and probe the corresponding impacts on functional RNAi.

To achieve this, we designed a series of *de novo* sequences with varied propensities for helical folding to occur with and without NAs. The sequences are cationic secondary amphiphiles with in-built cell-penetrating properties^11^. As a biological target we chose RNA interference (RNAi). This is a highly selective tool to knock down or silence gene expression with a clear therapeutic potential^12^. However, small interfering RNA (siRNA), which mediate RNAi by engaging with mRNA^13^, are particularly prone to degradation^14^, cannot cross cell membranes^15^ and require constant protection and a reliable means for intracellular delivery^16^.

For these reasons, RNAi is deemed specific as a performance test for the design series, comprising two subgroups – helices that co-fold with siRNA, i.e. responsively folded, and helices that fold without siRNA, i.e. autonomously folded.

## Results

### Responsively folded helices

**Figure 1:**
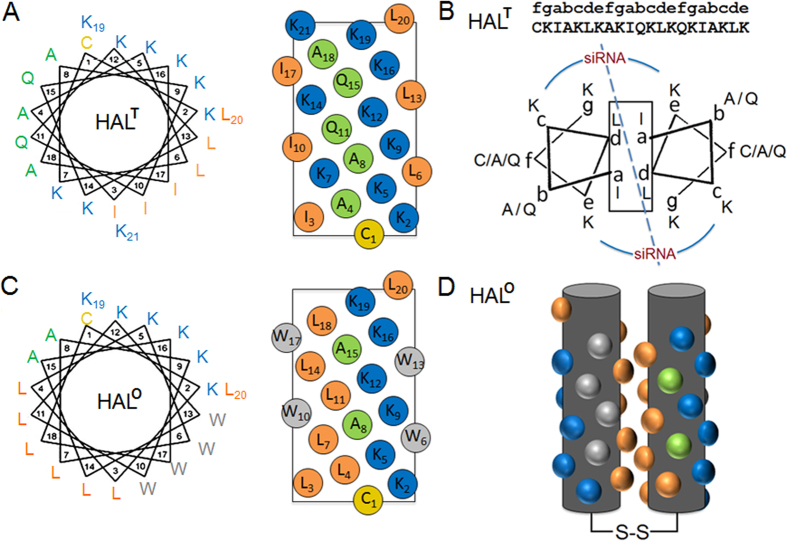
Peptide design. (**A**) Template HAL sequence (HAL^T^) and (**C**) HAL° sequence, configured on helical wheels (left) and helical nets (right) with 3.6 residues per turn. (**B**) A schematic for the amphipathic locking of HAL^T^ helices co-folded with siRNA and configured into coiled-coil helical wheels (3.5 residues per turn). The sequence is aligned with the *gabcdef* heptad repeat pattern. The dashed and semi-circular lines represent sites of siRNA binding. (**D**) A schematic for autonomously folded (pre-folded) HAL° helices depicted as helical cylinders. The leucine residues interface with the formation of a hydrophobic core. Key: residues for H, C, N and W faces are shown in orange, blue, green and grey, respectively. Cysteine is in yellow.

Our design rationale derives from a recognition that it is the extent to which peptides pre-fold in solution before interacting with siRNA that enables RNAi. To that effect the peptides were designed with increasing propensities for helical folding in solution. The sequences incorporate three amino-acid types in an order supporting a generic heptad CHNCHCN, where C is cationic, H is hydrophobic and N is neutral and polar (Table S1). For the purpose of this study, the sequences are termed helical amphipathic lockers (HAL). In a template sequence – HAL^T^ – the three amino-acid types alternate to avoid stretches of same types and form three corresponding faces – C, H and N (Fig. 1A and Fig. S1). Cationic lysines occupy C, H comprises alternating isoleucines and leucines to favour low oligomers^17^, whereas neutral glutamines and alanines compose N (Fig. 1A). The C face was made sufficiently large to bind NA and facilitate egress into the cytoplasm from acidified endosomes mimicking viral strategies relying on membrane-disrupting α-helical domains^18^. N, which does not contribute to any of these two functions, extends the polar face to balance the H face without compromising amphipathicity, while maintaining peptide solubility^19^. Finally, an N-terminal cysteine was added to the sequence to stabilize the formed helical interfaces via disulphide bonds and increase transfection efficiency^20^. The sequence is meant to internalize live cells via endocytosis following electrostatic binding to weakly anionic cell surfaces^21^.

### Peptide-siRNA co-folding promotes transfection but prevents RNAi

**Figure 2:**
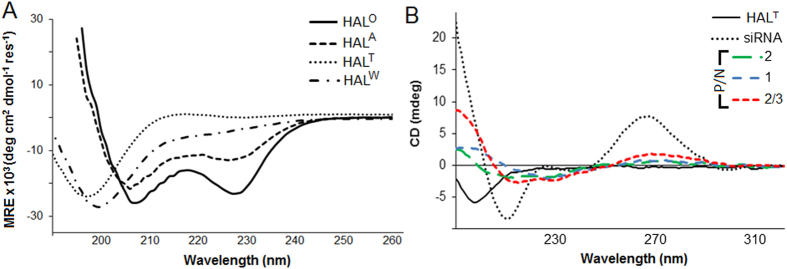
Peptide folding without and with siRNA. (**A**) CD spectra for HAL peptides (100 μM) in 10 mM MOPS at pH 7.4, 20 °C. (**B**) Raw CD spectra for HAL^T^, siRNA and HAL^T^/siRNA complexes at different P/N charge ratios. Peptide at constant 100 μM, 10 mM MOPS at pH 7.4 and 20 °C.

Consistent with the design, circular dichroism (CD) spectroscopy revealed that HAL^T^ did not fold in solution, but did with siRNA (Fig. 2). The peptide successfully transported siRNA (labelled with Alexa Fluor 647, AF647) into TREx-293 and HeLa cells with apparent punctuate staining patterns characteristic of an endocytic uptake^22^ (Figs S2A and S3A). As gauged by flow cytometry, the uptake increased with increasing peptide/siRNA (P/N) charge ratios for both cell lines (Figs S2B and S3B,C). Quantitatively, however, it remained comparable or lower than that for commercial transfection reagents, Lipofectamine^®^ RNAiMax (Lipo)^23^ and N-TER^TM ^24, used as positive controls (Figs S2B and S3B). As a consequence, no gene silencing was detected for HAL^T^-mediated anti-GFP siRNA in GFP-expressing Flp-In TREx-293 cells (Fig. S4A). The results may be explained by the lacking ability of the peptide to lyse endosomal membranes, which would require stronger peptide-lipid interactions. In this regard, viral fusion peptides and their hybrids, shown to act as pH-dependent endosomal releasing agents, often contain tryptophan pairs spaced at the helical *i, i* + *4* spacings^25^. Such an arrangement ensures the exposure of a tryptophan pair on one side of the folded or folding amphiphile allowing it to anchor to phospholipid membranes. It also seeds a hydrophobic interface owing to favorable π-π stacking interactions between indole rings^26^,^27^. With this in mind, the hydrophobic face of HAL^T^ was mutated with one *i, i* + *4* tryptophan stretch to give HAL^W^ (Fig. S1). The peptide folded in a similar manner to its precursor with elements of a helical structure and gave consistent increases in transfection (Figs 2, S2 and S3). However, it had no impact on gene silencing (Fig. S4A).

These findings are consistent with the results of earlier studies using cell-penetrating peptides (CPPs) and are important for two reasons. Firstly, CPPs translocate in an energy-dependent manner, while endosomal acidification appears to be a necessary prelude to cytoplasmic delivery^28^. Indeed, sodium azide and deoxyglucose, which together inhibit ATPases, completely blocked the entry of HAL^T^/siRNA into the cells which is indicative of energy-dependent translocation (Fig. S4B). Further, incubations of the cells with NH_4_Cl, which is used to block endosomal acidification, and hence escape, showed no apparent differences in median AF647 fluorescence versus that of control cells (NH_4_Cl-untreated) suggesting that HAL^T^/siRNA may remain trapped in endosomes (Fig. S4B). To partly arrest the endocytic uptake of HAL^T^/siRNA, while inhibiting endosomal acidification, transfections were treated with bafilomycin A1 (Baf A1)^29^. This antibiotic is a specific inhibitor of vacuolar ATPase proton pumps, which regulate sub-membranous pH and the formation of macropinosomes^30^. Baf A1 caused up to 50% reductions in the uptake of the complex compared to the control incubations implying that endosomal both uptake and escape were inhibited (Fig. S4B). Collectively, the results indicate that HAL^T^/siRNA uses an energy-dependent endocytic pathway to enter cells with possibly a limited capacity to escape endosomes.

The latter relates to the second reason. Although CPPs are capable of delivering siRNA into the cells with notable transfection efficiencies, these do not translate into an appreciable gene knockdown^31^. In order to understand if endosomal entrapment or fusion to lysosomes restricted gene silencing, the same transfections were performed in the presence of lysosomotropic chloroquine. The reagent gave only negligible improvements in GFP knockdown (Fig. S5), suggesting that siRNA remained trapped in the complexes. The effect appeared to be in synergy with the apparent lack of endosomal escape. Indeed, both CPPs and the HALs failed to enable RNAi. However, CPPS are not necessarily amphipathic or folded, while the HALs are amphipathic and fold only upon binding to siRNA.

### Autonomously folded helices

Based on this reasoning, the H faces of HALs appear to interface as the peptides co-fold with siRNA resulting in the clamping of the siRNA duplex. The scenario is akin to that of eukaryotic DNA topoisomerase 1 (Top1) locking duplex DNA^32^ or helix-turn-helix clamps regulating viral NA polymerases^33^. Top1 clamps inhibit DNA rotation and change DNA morphology to the extent sufficient to cause cell death. To lock, the HAL sequences accommodate a slight left-handed twist, which enables an interfacial burial of the H faces (Fig. 1B). This property owes to the heptad repeats that support the coiled-coil type of inter-helix locking^34^. Electrostatic interactions between the C face of HALs and siRNA together with cysteine bridges cement the structure further. As in the case of Top1, such clamping can be inhibited, though at the expense of NA-complexation. Therefore, an evident alternative is to pre-fold HALs into helical oligomers that would have sufficiently large cationic surfaces to complex NAs and an already buried hydrophobic interface (Fig. 1B).

To achieve this, another HAL was designed to fold autonomously, but with the formation of three distinct faces. A tryptophan face (W) comprising two tryptophan *i, i* + *4* spans was introduced between the H and C faces of the folded HAL^T^. This new face provides contiguous π-π stacking interactions shown to promote helical folding in solution^26^,^27^. The face also is expected to engage with endosomal membranes in a manner characteristic of membrane proximal anchors in viral transmembrane proteins^25^. Given that tryptophans and isoleucines impose similar helical penalties^35^, leucine and alanine, which have the highest helical propensities^36^, were used to re-balance the H and N faces, respectively. The resulting autonomously folded peptide, dubbed HAL^A^ (Fig. S1) was appreciably helical in solution featuring also a red-shifted minimum centred at 226 nm (Fig. 2A). The shift is characteristic of superposed aromatic bands indicating a constrained chiral environment for tryptophans thus confirming their *i, i* + *4* helical arrangement^36^. The ability of HAL^A^ to fold manifested in a notable increase in helicity, from 5% for HAL^W^ to 30% for HAL^A^ (Fig. 2A and Table S1)^37^, suggests an interplay between the W and H faces in setting-up hydrophobic cores. Indeed, both are presented by two contiguous *i, i* + *4* helical stretches that can interface with one another stabilising an α-helical structure, and both or either can be extended with an additional stretch to make the super-helix much stronger. Because leucine is more helical, but less hydrophobic, than tryptophan^38^, the H face was extended with another leucine pair by replacing two adjacent alanyl residues (Fig. 1C, Table S1 and Fig. S1). Any further hydrophobic extensions to this sequence would result in a polar angle (160°) being close to those of haemolytic peptides (≤120°)^39^. Therefore, to avoid likely cytotoxic scenarios this sequence completes the series as an optimal HAL or HAL° (Fig. 1C and Fig. S1). The peptide folded with a doubled helical content (65%) compared to that of HAL^A^, while showing similar spectral features (Fig. 2A). Although it is not deemed straightforward to reveal the exact nature of the oligomers, a helix-bundle type of folding with a limited capacity for propagation is likely to be favoured with the formation of amorphous nanoparticles. In accord with this, transmission electron microscopy (TEM) revealed 20–40 nm aggregates for HAL° and marginally larger nanoparticles (≤55 nm) with a median diameter of 33 ± 11 for HAL°/siRNA (Fig. S6).

### Peptide pre-folding promotes RNAi

**Figure 3:**
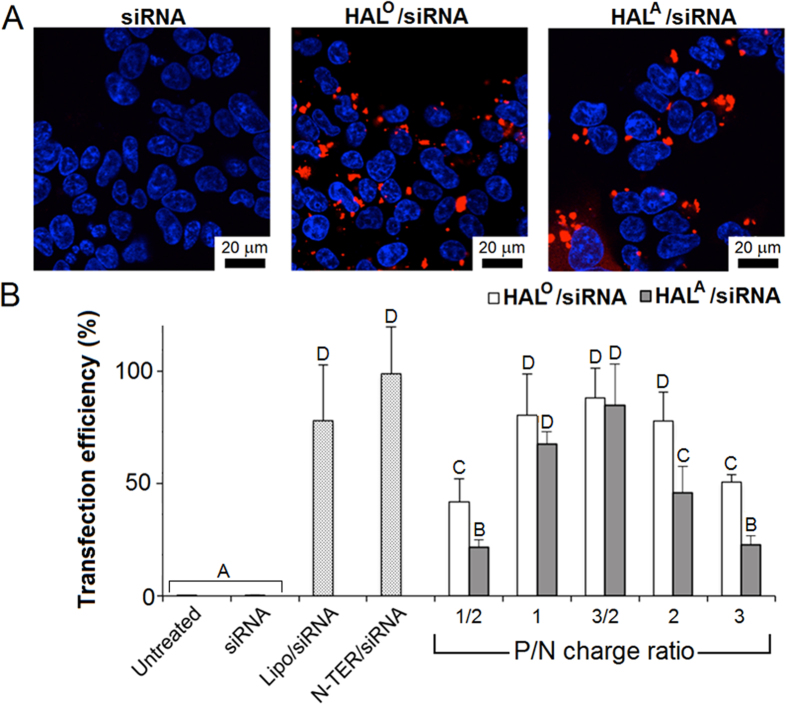
Cellular uptake of HAL/siRNA complexes. (**A**) Confocal microscopy images of Flp-In T-REx-293 cells incubated with AF647-siRNA, bare and complexed with HAL° and HAL^A^ at a 3/2 charge ratio for 2 h at 37 °C. Key: AF647-siRNA is red. Nuclei are stained with H33342 (blue). (**B**) Uptake of AF647-siRNA in Flp-In T-REx-293 cells measured by flow cytometry following incubations with HAL/AF647-siRNA complexes at different charge ratios, and AF647-siRNA complexed with commercial transfection reagents N-TER^TM^ and Lipofectamine^®^ RNAiMax. Transfection efficiencies were calculated using the median fluorescent intensity and expressed in percentage (the highest transfections by N-TER taken as 100%). Error bars denote standard deviation of three replicates. Letters denote key comparison and statistically different groups analysed by one-way ANOVA and Tukey’s test (p < 0.05).

**Figure 4:**
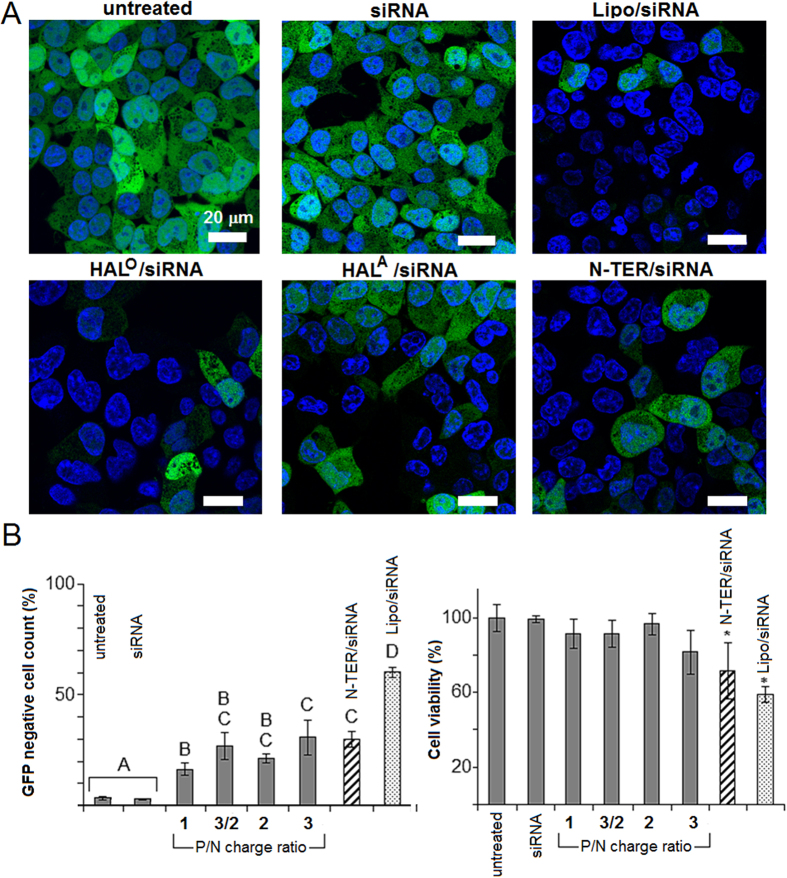
GFP-silencing and cell viability. (**A**) Confocal fluorescent micrographs of Flp-In T-REx-293 cells stably expressing GFP incubated with siRNA, bare and complexed with HAL° at a 3/2 charge ratio, HAL^A^ at a 3/2 charge ratio, N-TER^TM^ and Lipofectamine^®^ RNAiMax (Lipo) for 48 h at 37 °C. Key: nuclei stained with H33342 (blue), GFP is green. (**B**) GFP silencing measured by flow cytometry (left) and cell metabolic activity measured by alamarBlue^®^ assay (right) in Flp-In T-REx-293 cells following 24-h incubations with HAL°/siRNA at different charge ratios. The silencing is expressed in percentage with the total cell counts of an untreated GFP-negative population taken as 0%, allowing for false positive samples. Cell viability is expressed in % with the viability of untreated cells taken as 100%. Error bars denote standard deviation of three replicates. Results were analysed using one-way ANOVA followed by Tukey’s test for multiple comparisons (p < 0.05). Different letters represent groups with statistically different means (*B, left*). (*) denotes groups with a mean statistically different from the control conditions (*B, right*).

Both pre-folded peptides mediated higher transfection efficiencies in Flp-In T-REx-293 cells than those for HAL^T^ and HAL^W^, which were also statistically comparable to those of the commercial reagents (Figs 3, S2 and S3). Optimal P/N ratios (charge) appeared to be at a 3/2 range (Fig. 3). At this ratio appreciable gene silencing was detected for 24-h and 48-h incubations (Fig. 4 and Fig. S7). Consistent with the increased helicity, HAL° exhibited superior performances over that of HAL^A^ (Figs 3 and 4 and Fig. S7). Transfections with control siRNA, not coding for any known human gene, and the peptides alone confirmed that the silencing was due to the anti-GFP siRNA, used for RNAi, as opposed to non-specific reactions (Fig. S8). Importantly, the knockdown was achieved with cells retaining metabolic activities over the entire incubation periods with the peptides. In contrast, transfections with Lipofectamine^®^ RNAiMax, which gave seemingly stronger silencing, and partly with N-TER^®^, which gave similar silencing, were accompanied with impaired cell viability^40^ (Fig. 4B and Fig. S7).

**Figure 5:**
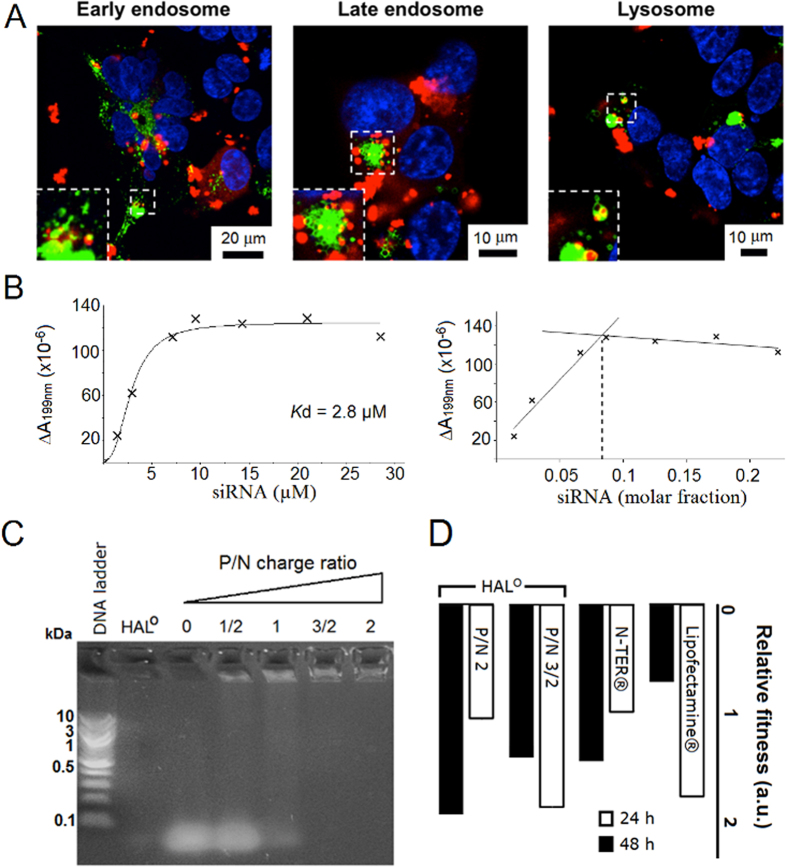
Endocytic trafficking and complexation of HAL°-siRNA. (**A**) Confocal fluorescent micrographs of FLp-In TREx-293 cells with stained endosomal vesicles incubated for 2 h with HAL°/siRNA at a 3/2 P/N charge ratio. CellLight^®^ endosomal and lysosomal GFP markers were used according to the proprietary protocols. Key: AF647-siRNA (red), nuclei stained with H33342 (blue), endosomes and lysosomes are stained with GFP (green). White dashed boxes are enlarged at the bottom left corner of each image. (**B**) Differential absorbance (∆A) at 199 nm versus the concentration and molar fraction of siRNA, derived from SRCD spectra. (**C**) Agarose gel (2% w/v) electrophoresis of HAL°/siRNA showing siRNA complexation as a function of P/N charge ratios. (**D**) Knockdown fitness of HAL° and commercial Lipofectamine^®^ RNAiMAX and N-TER^®^ (positive controls) normalised against siRNA alone (negative control) and the total counts of viable cells at different N/P (siRNA-peptide) molar ratios.

Similar to HAL^T^ and HAL^W^, transfections with the peptides gave punctate staining patterns (AF647) in perinuclear regions supporting an endocytic pathway (Fig. 3A). Since endosomes migrate towards the nucleus as they mature into late endosomes and lysosomes^41^, it was appropriate to probe the intracellular trafficking of the complexes using endosomal tracing markers. Co-transfections with CellLight^®^ reagents (baculovirus expressing GFP fused with endosomal and lysosomal proteins) revealed that siRNA (red) co-localised with the markers (green) in early endosomes with a substantial release of siRNA into the cytoplasm at later stages and negligible co-localisation in lysosomes (Fig. 5A). These observed differences are in good agreement with the GFP-silencing data and indicate that the peptides effectively escape endosomes delivering siRNA in quantities sufficient for loading into the RNA-induced silencing complex (RISC)^42^. The findings together with the folding, cell viability and TEM data also suggests that the peptides should bind and complex siRNA in a cooperative manner, which is a pre-requisite for avoiding the aggregation of complexes that otherwise may lead to cytotoxicity.

To gain a better insight into the nature of the complexation, changes in HAL°/siRNA folding at different siRNA concentrations were analysed by synchrotron radiation circular dichroism (SRCD) spectroscopy^43^. SRCD can provide information inaccessible to conventional CD, and allows for the extraction of residual CD spectra, with an improved signal to noise ratio, which are independent of overlapping signals from peptide, siRNA and their complexes. Differential absorbance (∆A) curves, obtained as a function of siRNA concentration and molar fraction (Fig. 5B and Fig. S9), returned the exponential parameter *n* at 2.5 indicating cooperative complexation, whereas HAL° affinity to siRNA (*Kd*, 2.8 μM) proved to be in the ranges expected for peptide/siRNA complexes^44^. The stoichiometry of the binding was at a siRNA molar fraction of 0.08, which corresponds to a 1.6 P/N charge ratio (Fig. 5B). This ratio closely matches the optimal transfection ratios at 3/2 (Figs 3b and 4b), at which siRNA complexation was essentially complete (Fig. 5C), and at which siRNA remained protected by peptide complexation from enzymatic degradation (Fig. S10). Such synergy between optimal transfection and complexation ratios (3/2) was consistent with equally effective gene silencing in HeLa. Specifically, HeLa cells containing two housekeeping genes, ACTB (β-actin, targeted) and GAPDH (reference)^45^, were transfected with HAL°/siRNA and the silencing of β-actin mRNA was quantitatively monitored by reverse transcription polymerase chain reaction (RT-PCR) at 24 ± 2 hours and 48 ± 2 hours post-transfection. HAL°/siRNA at the ratio revealed comparatively higher levels of knockdown than those of the commercial reagents used (Fig. 5D). The levels were expressed relative to cells treated with siRNA alone (background) and normalised against viable cell counts to give relative knockdown fitness^46^. Similar to the results obtained for GFP silencing in Flp-In T-REx-293 cells, no apparent increases were observed for the peptide at higher P/N ratios (Fig. 5D). Thus, both knockdown efficiencies and the optimal P/N ratio were found to correlate for the two unrelated cell lines, independent knockdown measurements and different gene targets.

## Discussion

Collectively, the results prompt a generic rationale for functional RNAi, according to which it is the inter-locking of helices prior to binding to siRNA that enables RNAi. The design of helical amphipathic lockers elaborated in this study differentiates autonomously folded helices, which promote gene silencing, from helices folded in response to RNA binding, which do not. Each of the helical designs complexes siRNA and delivers it into cells via energy-dependent endocytosis. Each can interface to oligomerize. However, only autonomously folded HAL^A^ and HAL° proved to support the synergistic inter-locking of hydrophobic interfaces without inhibiting siRNA release and thence gene silencing. HAL^T^ and HAL^W^ lock on the siRNA they bind, acting as helical clamps^33^. In contrast, HAL^A^ and HAL° exhibit an additional W face, which cements folded helices into nanoparticles creating thus large cationic surfaces. These nanoparticles bind siRNA and permeabilise endosomal membranes as masked endosomolytic agents^47^,^48^. This latter property induced by membrane binding is characteristic of folding-responsive antimicrobial peptides^49^ and amphipathic helical domains of viral capsid proteins^18^ that lyse microbial and endosomal membranes, respectively. More specific naturally occurring systems strictly regulate the extent to which hydrophobic faces are involved in interfacial contacts. For example, in the DNA-binding helical regions of transcription factors charged one-face interactions dominate, while the hydrophobic face is less pronounced allowing excluding excessive or competing non-polar interactions. In this light, the introduced concept of helical lockers presents a structure-function rationale of balanced amphipathic scaffolds that can help better understand and exploit substrate-independent delivery of nucleic acids.

## Methods

### Peptide Synthesis and Purification

All peptides were assembled on a Rink amide MBHA resin using standard Fmoc/^t^Bu solid-phase protocols with HBTU/DIPEA as coupling reagents on an automated Liberty microwave peptide synthesiser (CEM Corp., USA). Capping via acetylation was performed on resin (90% DMF, 5% acetic anhydride, 5% pyridine), followed by cleavage from the resin (95% TFA, 2.5% TIS, 2.5% EDT) and purification by reversed phase high performance liquid chromatography (RP-HPLC). The identity of each peptide was confirmed by analytical RP-HPLC and MALDI-ToF MS on an Autoflex III (Bruker, Germany) (Supplemental Table S1). Analytical and semi-preparative RP-HPLC was performed on a JASCO HPLC system (PU-980; Tokyo, Japan) using a Vydac C18 analytical and semi-preparative (both 5 μm) columns (Grace, USA). Both analytical and semi-preparative runs used a 10–70% B gradient over 30 min at 1 mL/min and 4.5 mL/min, respectively, with detection at 280 and 220 nm (buffer A, 5% and buffer B, 95% aqueous CH_3_CN, 0.1% TFA). The purified peptides were lyophilised and stored at −80 °C until use. Stock solutions were prepared from lyophilised peptides dissolved in milli-Q water containing 2% v/v DMSO.

### siRNA

The siRNA (anti-GFP siRNA and the negative control siRNA) used in this study were obtained from Eurogentec (Belgium). The Alexa Fluor 647 (AF647) labelled siRNA (AF647-siRNA) was also obtained from Eurogentec (Belgium) and had the following sequence: AF647-GCAAGCUGACCCUGAAGUUCTT-3′ (sense strand) and GAACUUCAGGGUCAGCUUGCTT-3′ (antisense strand). siRNA stock solutions were prepared in RNase-free water at 100 μM.

### Preparation of Peptide/siRNA Complexes

Peptide stock solutions (1.0 mM) were diluted in either 10 mM MOPS at pH 7.4 for CD and TEM experiments, or in 1x PBS at pH 7.4 for agarose gel electrophoresis and transfection experiments, and at different concentrations to keep the final volume of complexes below 25 μl. The peptide dilutions were added to 30 pmol siRNA and mixed by pipetting. The quantity of siRNA was kept constant at 30 pmol while that of peptide was varied to adjust P/N charge ratios in the range of 0.5:1 to 10:1. The peptide/siRNA solutions were incubated for 30 min at room temperature to ensure complexation.

### Agarose Gel Electrophoresis

#### siRNA complexation as a function of P/N charge ratios

Agarose gel electrophoresis of peptide/siRNA complexes of different P/N charge ratios was performed in 2.0% (w/v) agarose in tris/borate/EDTA buffer containing 0.5 μg/ml ethidium bromide. The gels were immerged in the buffer and subjected to a voltage of 75 V for 45 min.

#### Nuclease protection assay as a function of P/N charge ratios

RNase A (2 μg) was added to peptide/siRNA complexes of different P/N charge ratios to a final concentration of 100 μg/ml. The obtained mixtures were then incubated for 30 min at room temperature followed by electrophoretic analyses in a 2% (w/v) agarose gel in tris/acetic acid/EDTA buffer containing 0.5 μg/ml ethidium bromide. The gels were immerged in the buffer and subjected to a voltage of 75 V for 45 min.

After completion, all gels were viewed under UV light and photographed by a U:Genius system (Syngene, USA).

### Circular Dichroism Spectroscopy

Circular dichroism spectra were recorded using electronic circular dichroism (ECD) while the analysis of peptide/siRNA complexes was performed using synchrotron radiation circular dichroism (SRCD) spectroscopy at beamline B23 of Diamond Light Source (Harwell, UK). Both ECD and SRCD measurements were performed at a fixed peptide concentration of 100 μM. Both set-ups were equipped with a Peltier temperature controller set to 20 °C, unless otherwise stated, and measurements were acquired using 1 nm step, 1 s collection time per step and 1 nm spectral band width. ECD spectra were recorded using a Chirascan Plus (Applied Photophysics, UK) spectropolarimeter (0.05 cm path length). SRCD spectra were recorded using an Olis CD spectropolarimeter (0.01 cm path length). SRCD measurements were performed for peptide/siRNA complexes prepared at a constant peptide concentration (100 μM) and varied siRNA concentrations (1.4–64.3 μM) to a fixed volume of 20 μL. All measurements were recorded in ellipticities, and after baseline correction, were converted to mean residue ellipticities by normalising for the concentration of peptide bonds. The percentage α-helicity of the peptides in solution was estimated as reported elsewhere (−100([*θ*]_222_ + 3000)/33 000 was used to calculate the percent helix)^37^. SRCD data from peptide/siRNA titrations were processed and analysed using CD*Apps*^50^. The fitting of the experimental data using the Hill equation was used to determine *K*d^51^.

### Transmission Electron Microscopy (TEM)

Droplets (8.0 μl) of peptide/siRNA complexes were deposited on glow discharge treated carbon coated copper grids (1 min) and dried by blotting using filter paper. The grids were negatively stained with filtered 0.75% uranyl acetate (8 μl, 10 s). Excess staining was removed with filter paper. The complex coated grids were imaged on a Tecnai G2 F20 (Fei, USA). Sizing of the aggregates and peptide/siRNA complexes was performed on the TEM images using ImageJ (NIH, USA).

### Cell Culture and Transfection

Flp-In T-REx-293 cells (Invitrogen, UK) stably expressing eGFP-C1 were donated by Dr Meng Lu and Prof. Alan Tunnacliffe and cultured as previously described^52^. Flp-In T-REx-293 cells were seeded in NUNC Lab-TEK coverglass chambered 4-well imaging plates for confocal microscopy or NUNC 4-well plates for flow cytometry at a density of 50,000 cells/well. The cells were incubated overnight at 37 °C and 5% CO_2_, in growth media containing 1 μg/mL tetracycline, to allow for cell attachment and GFP production. Prior to transfection, growth media was removed from each well and replaced with 200 μL Opti-MEM. The complexes formed as previously described with peptide and AF647-siRNA or anti-GFP siRNA or negative control siRNA at different charge ratios were added to each well. Lipofectamine^®^ RNAiMax (Invitrogen, UK) and N-TER^TM^ (Sigma, UK) were used as positive controls according to proprietary protocols. Control wells of cells in Opti-MEM (untreated) and cells with 30 pmol siRNA in solution (siRNA) were also prepared. Cells were incubated for 3 h at 37 °C under 5% CO_2_. After incubation, the Opti-MEM solutions were discarded and replaced with 500 μL growth media containing 1 μg/mL tetracycline in order to maintain a steady and continuous production of GFP mRNA. The cells were further incubated at 37 °C under 5% CO_2_ for 24 h and 48 h. Transfection efficiencies were expressed as a function of median absorbance and expressed in percentage (the highest transfections taken as 100%).

### Poisoned Transfection

Growth media was removed from wells containing Flp-In T-REx-293 cells after overnight incubation allowing for cell attachment. 200 μL Opti-MEM pre-incubated at 37 °C containing either just Opti-MEM (control samples), 10 mM sodium azide and 50 mM deoxy-glucose (sodium azide samples), 50 mM ammonium chloride (NH_4_Cl samples), or 200 nM bafilomycin A1 (Baf A1 samples) were added to cells. These samples were further pre-incubated for 30 min at 37 °C under 5% CO_2_. After pre-incubation, peptide/AF647-siRNA complexes were added to each well and incubated for 2 h at 37 °C under 5% CO_2_.

### Localisation of Peptide/siRNA complexes in the Endosomal Pathway

Growth media was removed from wells containing Flp-In T-REx-293 or HeLa cells after overnight incubation allowing for cell attachment. CellLight^®^ Early Endosomes-GFP (30 μL), Late Endosomes-GFP (25 μL) or Lysosomes-GFP (30 μl, all from Invitrogen, UK) were added to different wells and the cells were incubated for another 16 h to allow for expression of the CellLight vectors. Transfection was performed as described above with peptide/AF647-siRNA complexes and the cells were incubated for 1.5–2 h at 37 °C under 5% CO_2_.

### Confocal microscopy

After 2-h, 24-h or 48-h incubations following transfection, the media was discarded and replaced with 500 μL growth media containing 1 μg/mL of H33342 to stain cell nuclei. The cells were further incubated for 20 min at 37 °C under 5% CO_2_ and then immediately imaged on a Leica TCS SP5 confocal microscope with an x63 oil lens.

### Flow Cytometry

Quantification of labelled siRNA uptake and GFP knockdown was performed by flow cytometry. After 2-h incubations with complexes for uptake studies, or 24-h and 48-h incubations for gene silencing studies, cells were trypsinized and re-suspended in 200 μL PBS. AF647 or GFP fluorescence were measured by flow cytometry on a Cytek DxP8 FACScan (USA) and analysed using FlowJo software (USA). For both uptake and knockdown studies, at least 10^4^ events were gated from each ≥10^5^ subset measured for each sample by forward scatter and side scatter on the 488-nm or 633-nm lasers to extract and analyse 6–9 × 10^3^ single viable cells (Figs S3C and S11). From the untreated control cells, incubated with 200 μL Opti-MEM containing no siRNA and no peptide, a GFP negative population was selected and taken as 0% negative, allowing for false positive samples (Fig. S11). This gate was then used on all other samples to measure the relative population of GFP negative cells, which was expressed in percentage.

### Cell Viability

Cell viability was measured using the alamarBlue^®^ assay (Invitrogen, UK) for Flp-In T-REx-293 cells incubated with peptide/siRNA complexes. Cells were seeded, transfected and incubated for 24 h or 48 h with Opti-MEM solutions containing various peptide/siRNA concentrations as described for the transfection methods above. The alamarBlue^®^ assay was then performed following the proprietary protocol (3 h incubation at 37 °C under 5% CO_2_), with total absorbance measured at 570 nm and 600 using a Spectrostar Nano-plate reader (BMG Labtech, Germany). Viability was calculated according to the proprietary instructions, allowing for spectral overlap, as a function of total absorbance and expressed in percentage (untreated cells taken as 100%).

### Transfection and gene knockdown measured by RT-qPCR in HeLa

HeLa cells were maintained in DMEM cell culture medium supplemented with serum growth supplement and antibiotics (gentamicin and amphotericin B) in 25 cm^2^ culture flasks, and grown at 37 °C, 5% CO_2_ for 24 hrs to reach 60% confluency. The cells were then washed (x3) with PBS and trypsinized followed by the addition of trypsin inhibitors to eliminate secondary toxic effects of trypsin. Detached cells were spun down by centrifugation, and the excess solvent was replaced by cell growth media. 10 μL of cell solution was mixed with 10 μL of Trypan blue. The mixture was then placed on a counting plate to count cells (25 × 10^3^ cells per well). Before transfection, the cells were washed (x3) with OptiMEM^®^-serum reduced media.

#### siRNA transfections for knockdown

mRNA concentrations in cells transfected with peptide/siRNA and controls were measured according to the MIQE guidelines^45^. Lipofectamine^®^ and N-TER^®^ were used as positive controls and prepared according to the proprietary protocols. The knockdown assay was performed using two recommended housekeeping genes, ACTB (targeted) and GAPDH (reference). siRNA alone was used as a negative (background) control. Proprietary primers (design optimised for PCR), RNA extraction and RT-qPCR kits together with method development protocols were adapted to limit assay optimisation. All measurements were done in triplicate.

β-actin siRNA (30 pmole) was used for transfection with peptide concentration adjusted to the desired P/N. Preparations with siRNA added into peptide after or before the assembly were similar. siRNA was incubated with peptide in MOPS (20 μL, 10 mM, pH 7.4, overnight) followed by incubations in OptiMEM^®^ (200 μL). Prepared peptide/siRNA or controls were added to cells and incubated for three hours. After the 3-hour incubations the cells were supplemented with complete DMEM media (20% serum, 200 μL). Further incubations were performed over 48 hours marking different time points. For RNA extraction, cells were harvested as described above and RNA was prepared using mini RNeasy mini^®^ prep kit. cDNA was prepared from the RNA using the QuantiTect reverse transcription kit and quantified using two-step RT-qPCR dual hybridization with a QuantiFast probe assay kit (all from Qiagen, USA). The PCR hydrolysis probes for β-actin and GAPDH labelled with FAM and MAX dyes were used as per the proprietary protocols (Qiagen).

Cells harvested from a single well were lysed (10^5^ cells in 350 μL of lysis buffer) and total RNA was purified (RNeasy mini^®^). RT was performed (QuantiTect^®^) in PCR 0.2 mL tubes on a GeneAmp PCR system 2700 (Applied Biosystems, UK) using 30–60 ng of total RNA according to the proprietary protocols. qPCR was performed on a SmartCycler^®^ using Software v2.0d (Cepheid). PCR of the diluted cDNA product (1–10 ng) was monitored over 45 cycles with the quantification cycle (C_q_) determined using a manual threshold of 30 fluorescence units. PCR titrations of both genes resulted in co-linear amplification. Control samples of no template control (contamination during qPCR) and no RT control (contamination by genomic DNA during RT) were negative (C_q_ > 35 cycles) indicating no measureable DNA contamination. Fitness levels were calculated based on the normalised function of cells treated with siRNA alone (negative control) and against the total counts of viable cells (cell viability):


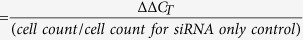


where ΔΔC_T_ is the knockdown efficiency of ACTB

- Relative to reference GAPDH gene for each transfection vector (ΔC_T_ (vector))ΔC_T_ (vector) = C_T_ (ACTB) − C_T_ (GAPDH), where the threshold cycle (C_T_) is calculated from the PCR thermal cycle. and
- To the siRNA only control (ΔC_T_ (siRNA only control));

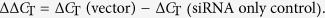


## Statistical Analysis

Statistical analysis for all the analytical data was performed by SPSS statistics version 23 using ANOVA followed by a Tukey’s post-test for three independent experiments. To allow comparison multiple means comparison tests were performed, with p values <0.05 considered statistically significant. The results are expressed as an average ± standard deviation. The Levene test was also performed in cases to assess the homogeneity of variance. The test was followed by the independent samples t-test for equality of means, with p values <0.05 considered statistically significant.

## Additional Information

**How to cite this article**: Guyader, C. P. E. *et al*. Autonomously folded α-helical lockers promote RNAi*. *Sci. Rep.* **6**, 35012; doi: 10.1038/srep35012 (2016).

## Supplementary Material

Supplementary Information
