# Supplementary material for: Autonomously folded α-helical lockers promote RNAi*
Source: Sci Rep. 2016 Oct 10;6:35012. doi: 10.1038/srep35012 (PMC5056365; doi:10.1038/srep35012)
Supplement: Supplementary Information [file srep35012-s1.pdf]

Autonomously folded α-helical lockers promote RNAi

Christian P. E. Guyader, Baptiste Lamarre, Emiliana De Santis, James E. Noble, Nigel K. Slater and Maxim G. Ryadnov

Supporting Information

Table and Figures

Table S1. Peptides used in the study

Name	Sequence^{a}	m/z		α-helicity, %^{b}
		calculated	measured	
HAL^{T}	CKIAKLKAKIQKLKQKIAKLK	2422.2	2422.3	0
HAL^{W}	CKIAKWKAKWQKLKQKIAKLK	2568.3	2568.7	5
HAL^{A}	ac-CKLAKWLAKWAKWLAKWLKL	2526.2	2526.4	30
HAL^{O}	ac-CKLLKWLAKWLKWLAKWLKL	2610.4	2610.6	65

^{a}peptide terminal amides; ^{b}calculated using the equation: $-100([\Theta]_{222} + 3000)/33\ 000$ (37)

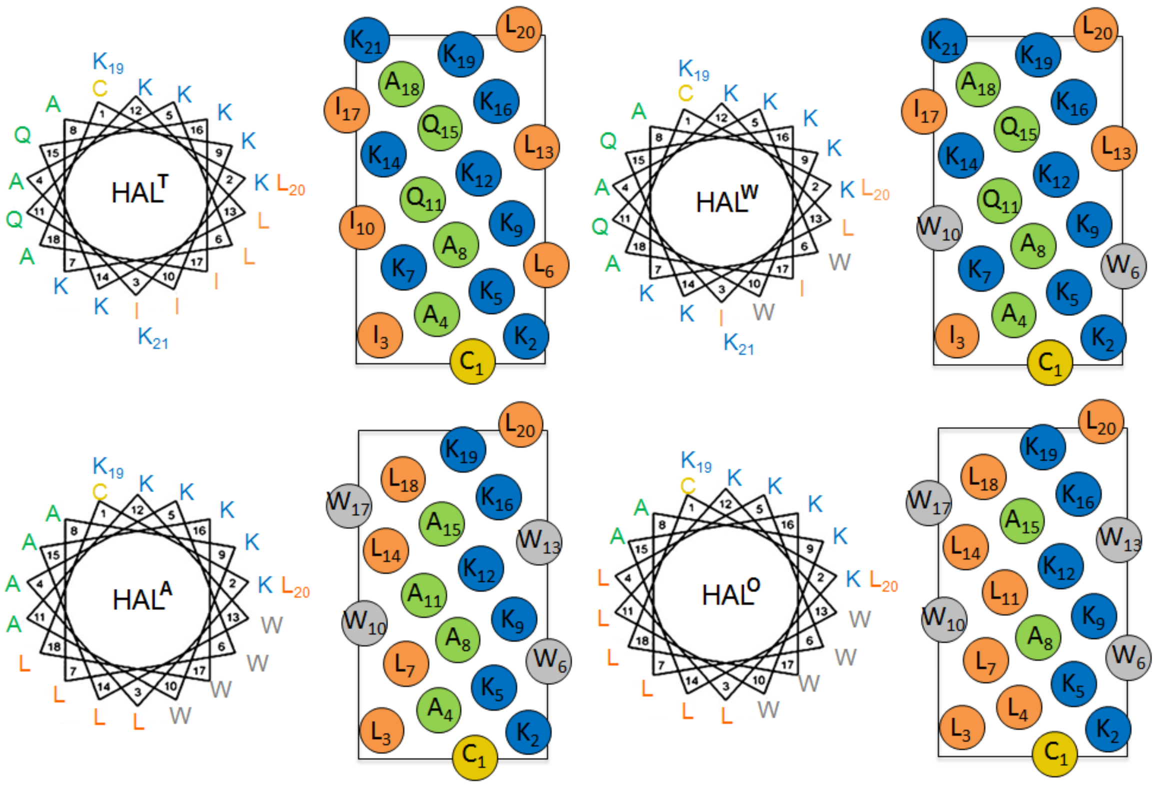

Figure S1. Peptide design. HAL sequences configured on helical wheels (left) and helical nets (right) with 3.6 residues per turn. Key: residues for H, C, N and W faces are shown in orange, blue, green and grey, respectively. Cysteine is in yellow.

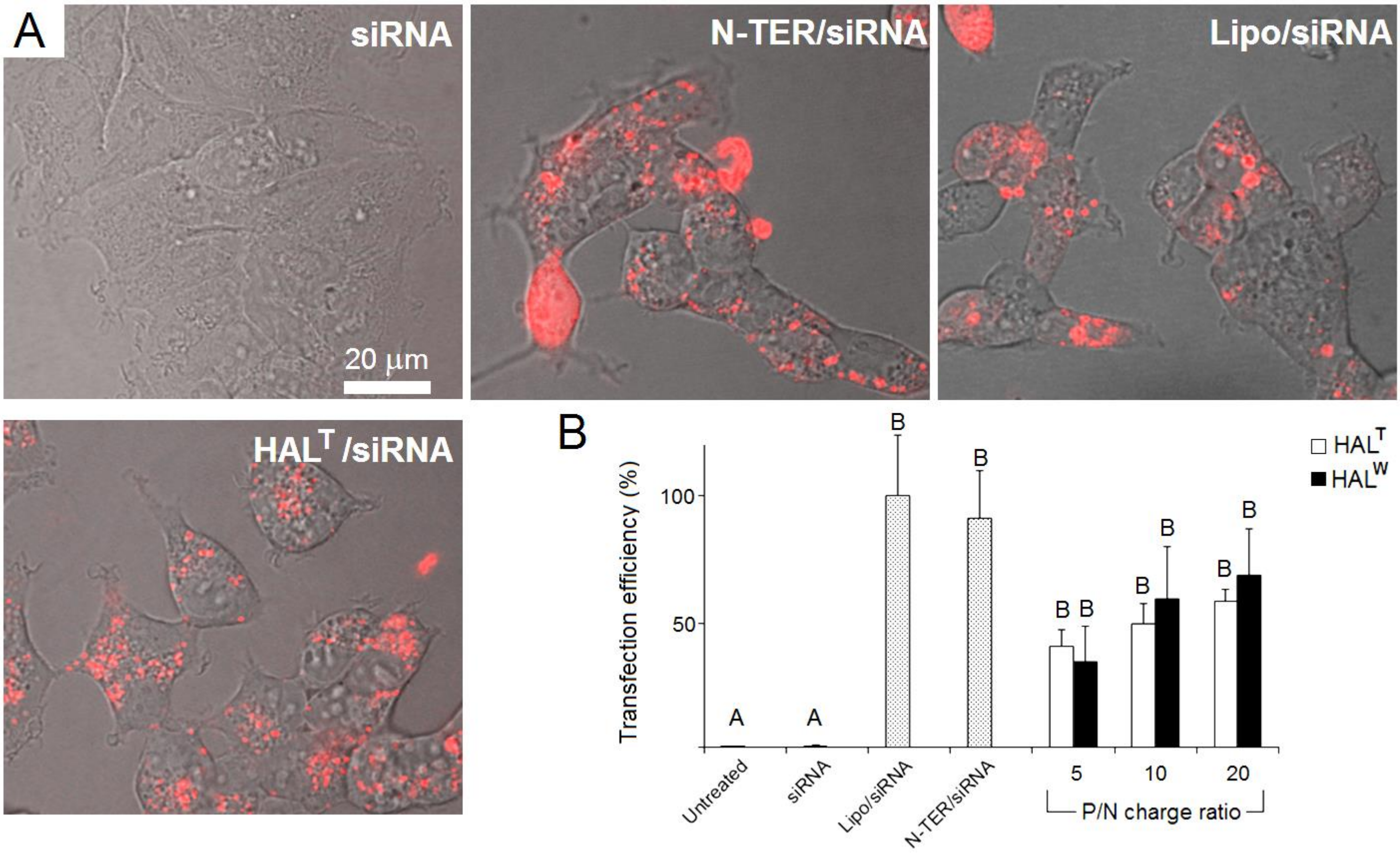

Figure S2. siRNA delivery into of Flp-In T-REx-293 cells. (A) Overlaid confocal fluorescence and bright field images of Flp-In T-REx-293 cells incubated with AF647-siRNA (red) complexed with HAL^T at a P/N charge ratio of 10:1 for 2 h at 37°C. Results are compared to the negative control of AF647-siRNA alone, and AF647-siRNA complexed with commercial transfection reagents N-TER™ and Lipofectamine® RNAiMax (Lipo), as positive controls. (B) siRNA uptake measured by flow cytometry of AF647-siRNA in 293-TREx cells incubated for 2 h at 37°C with HAL^T/AF647-siRNA and HAL^W/AF647-siRNA complexes at different P/N charge ratios. Results are compared with those for commercial transfection reagents N-TER™ and Lipofectamine® RNAiMax (Lipo) complexed with AF647-siRNA according to the proprietary protocols. Transfection efficiencies were calculated using the median fluorescent intensity of the samples and expressed in percentage (the highest transfections by Lipo taken as 100%). Error bars denote standard deviation of three replicates. Letters denote statistically different subsets analysed by one-way ANOVA followed by a Tukey's test ($p < 0.05$).

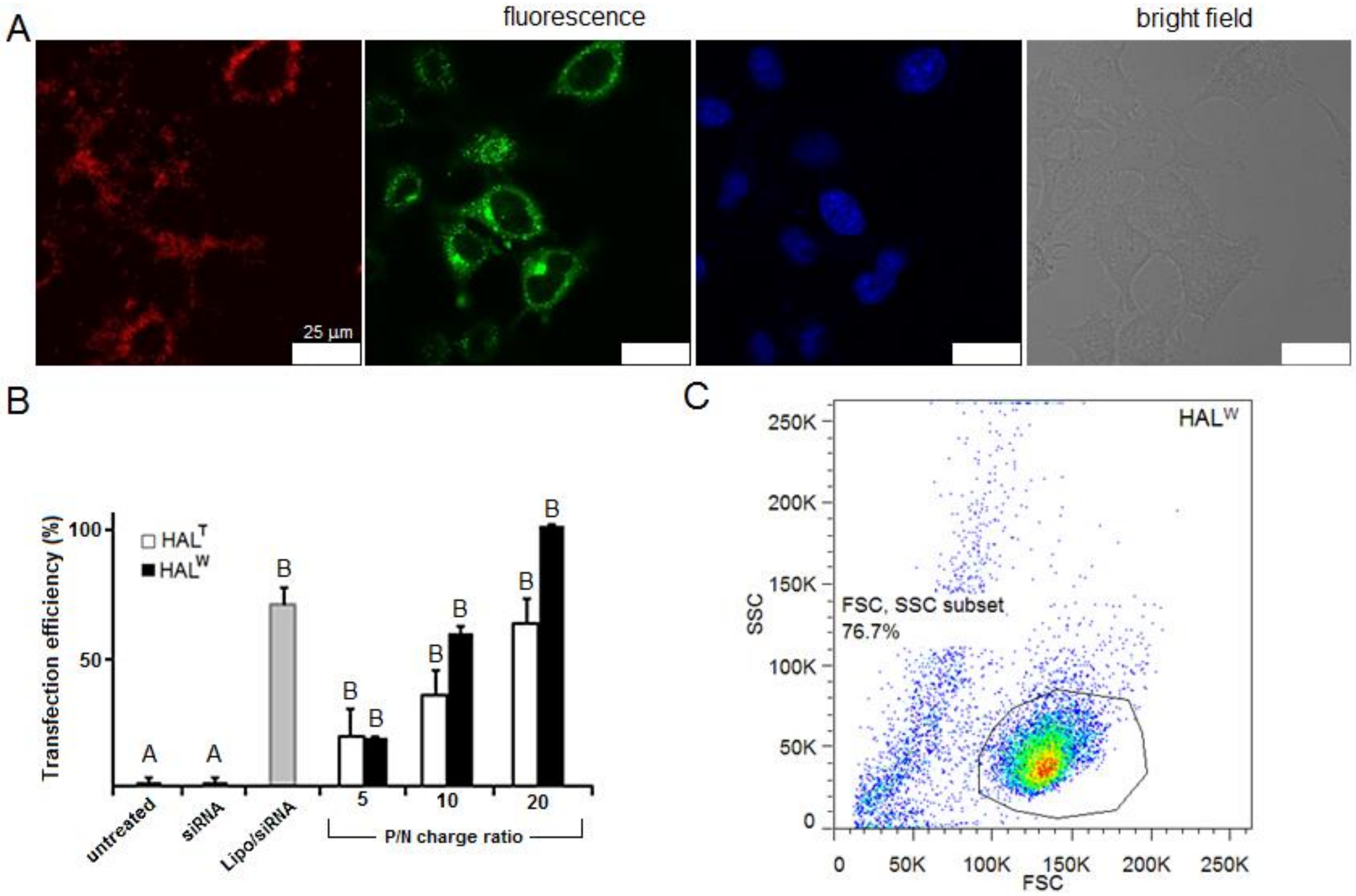

Figure S3. siRNA delivery into HeLa cells. (A) Confocal fluorescence and bright-field images of HeLa cells incubated with AF647-siRNA (red) complexed with HAL^T at a P/N charge ratio of 10:1 for 1.5 h at 37°C. Key: AF647-siRNA is red. Nuclei are stained with H33342 (blue), endosomes are stained with CellLight® (green). (B) siRNA uptake measured by flow cytometry of AF647-siRNA in HeLa cells incubated for 1.5 h at 37°C with HAL^T/AF647-siRNA and HAL^W/AF647-siRNA complexes at different P/N charge ratios. Results are compared to the negative control of untreated cells, AF647-siRNA alone and AF647-siRNA complexed with the commercial transfection reagent Lipofectamine® RNAiMax (Lipo) as a positive control. Transfection efficiencies were calculated using the median fluorescent intensity of the samples and expressed in percentage (the highest transfections by HAL^W at P/N 20 charge ratio was taken as 100%). Error bars denote standard deviation of three replicates. Letters denote statistically different subsets analysed by one-way ANOVA followed by a Tukey's test ($p < 0.05$). (C) Flow cytometry charts of cell population gating: at least 10^4 events were gated (circled in the charts) from each $\geq 10^5$ subset measured for each sample by forward scatter and side scatter (X and Y axis, respectively) on the 633-nm laser to extract and analyse single viable cells.

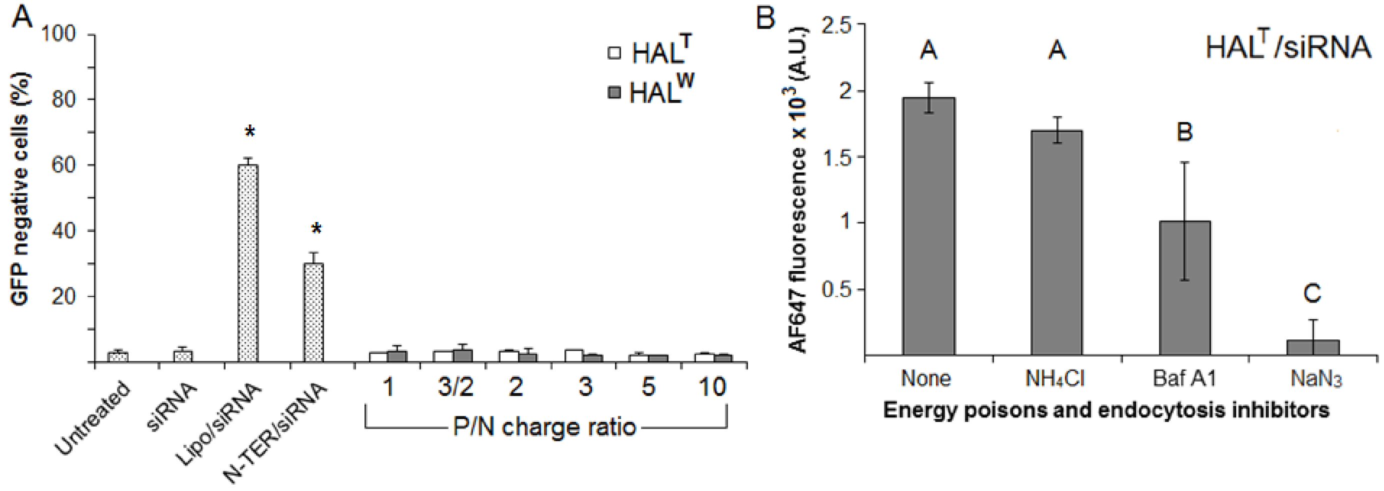

Figure S4. RNAi and inhibited siRNA delivery. (A) GFP silencing in Flp-In T-REx-293 cells measured by flow cytometry following 24-h incubations at 37°C with HAL^T/AF647-siRNA and HAL^W/AF647-siRNA at different P/N charge ratios at 30 pmol siRNA. Results are compared with those for commercial transfection reagents N-TER™ and Lipofectamine® RNAiMax (Lipo) complexed with AF647-siRNA according to the proprietary protocols. Error bars denote standard deviation of three replicates. (B) Median AF647 fluorescence measured by flow cytometry indicating the uptake of peptide/siRNA complexes at a P/N charge ratio 10 in Flp-In T-REx-293cells incubated with endosomal and energy inhibitors: 50 mM NH_4Cl, 200 nM bafilomycin A1 (Baf A1) and 10 mM NaN_3 with 50 mM deoxy-glucose. Error bars represent the standard deviation of three replicates. Results were analysed using one-way ANOVA followed by Tukey's test for multiple comparisons ($p < 0.05$). (*) denotes groups with a mean statistically different from the control conditions (*A*). Different letters represent groups with statistically different means (*B*).

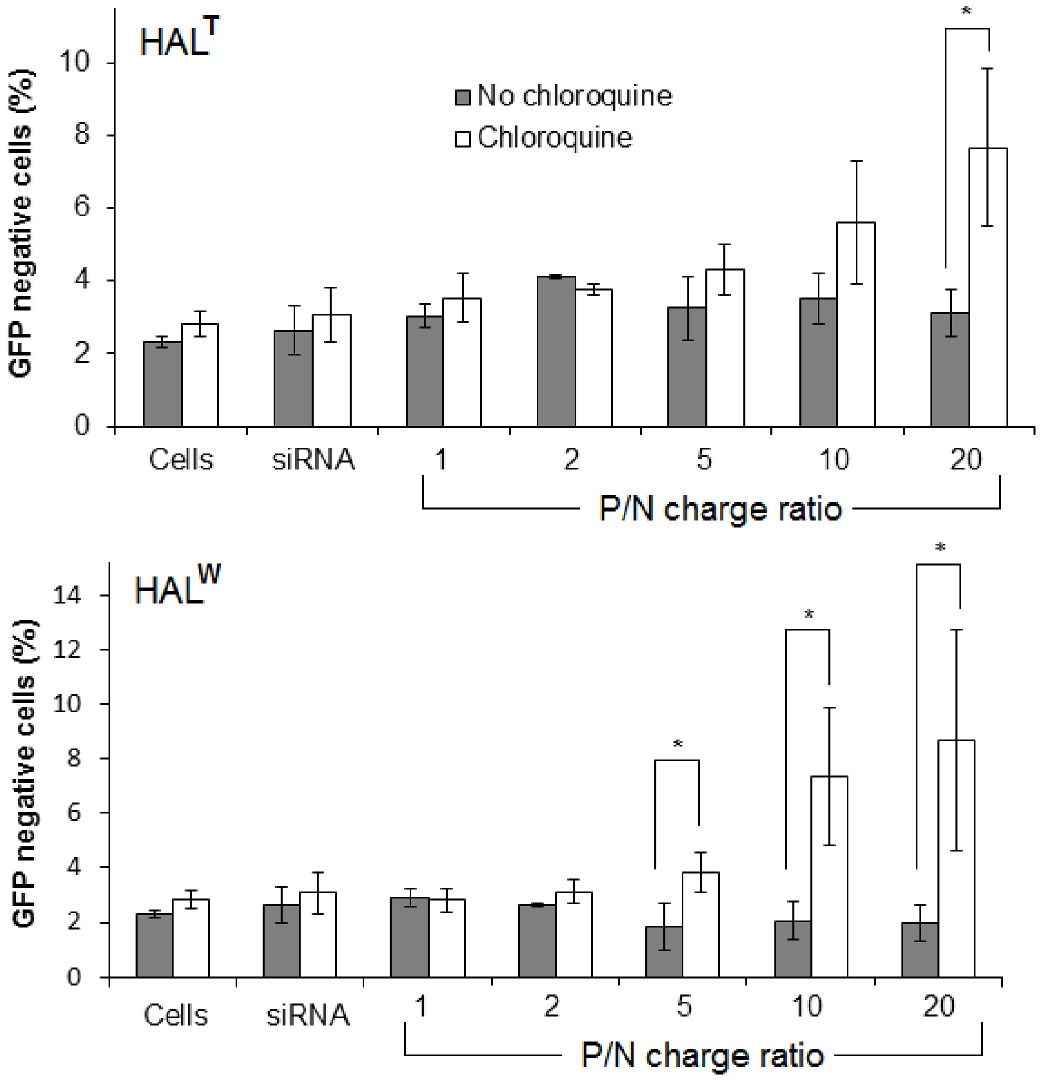

Figure S5. Effect of the lysosomotropic agent chloroquine (100 μM) on GFP silencing for Flp-In T-REx-293 cells transfected with HAL^T/siRNA (A) or HAL^W/siRNA (B). GFP knockdown was measured by flow cytometry following 48-h incubations at 37°C (3-h in the presence of peptide/siRNA complexes and 100 μM chloroquine in Opti-MEM followed by 45-h in growth media containing no chloroquine), and expressed in percentage with the total cell counts of an untreated GFP-positive population taken as 0%, allowing for false positive samples. Error bars denote standard deviation of three replicates. * denotes statistically different means using the independent samples *t* test ($p < 0.05$).

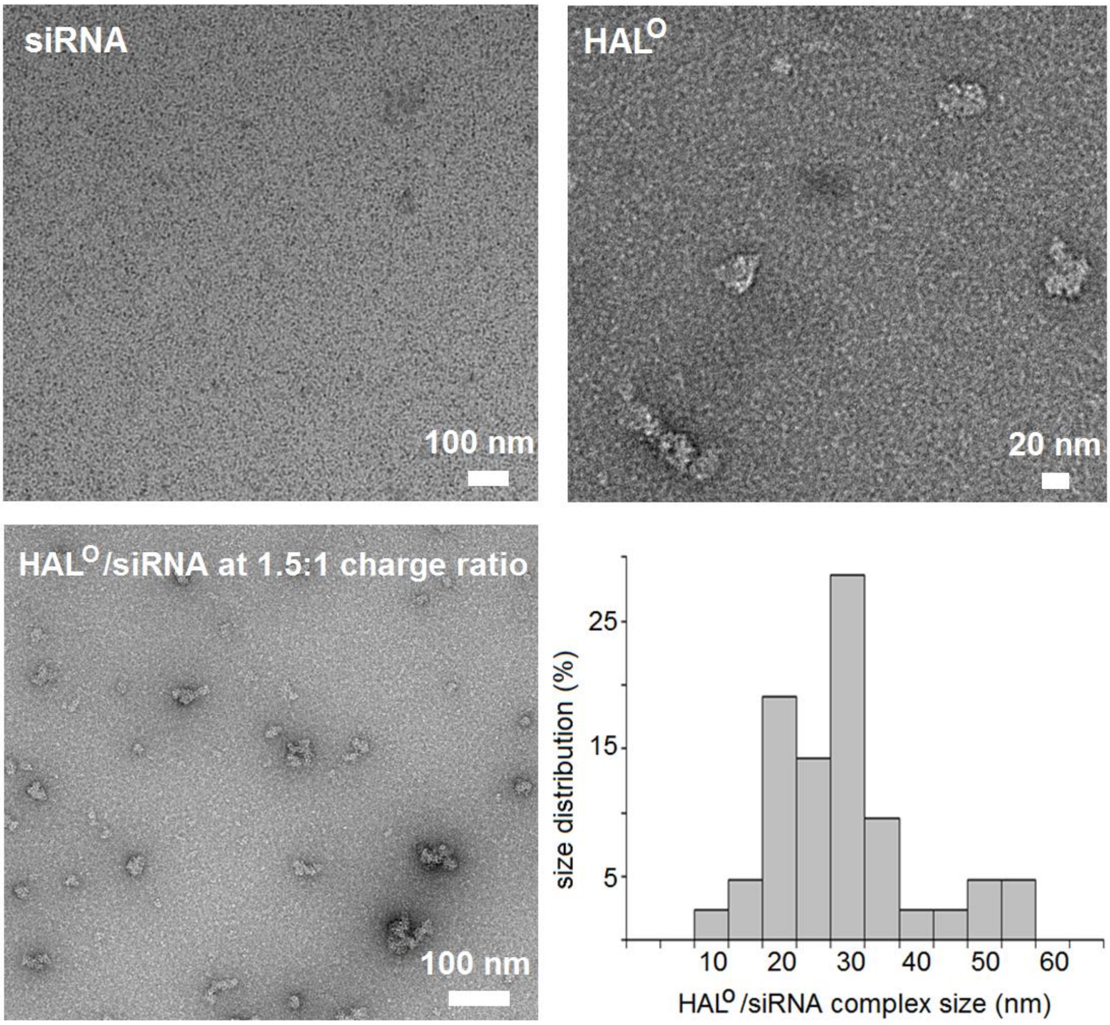

Figure S6. Electron micrographs of siRNA, HAL^{O} and HAL^{O}/siRNA and the size distribution of HAL^{O}/siRNA at a 3/2 (charge) P/N ratio calculated for 40 over particles by ImageJ.

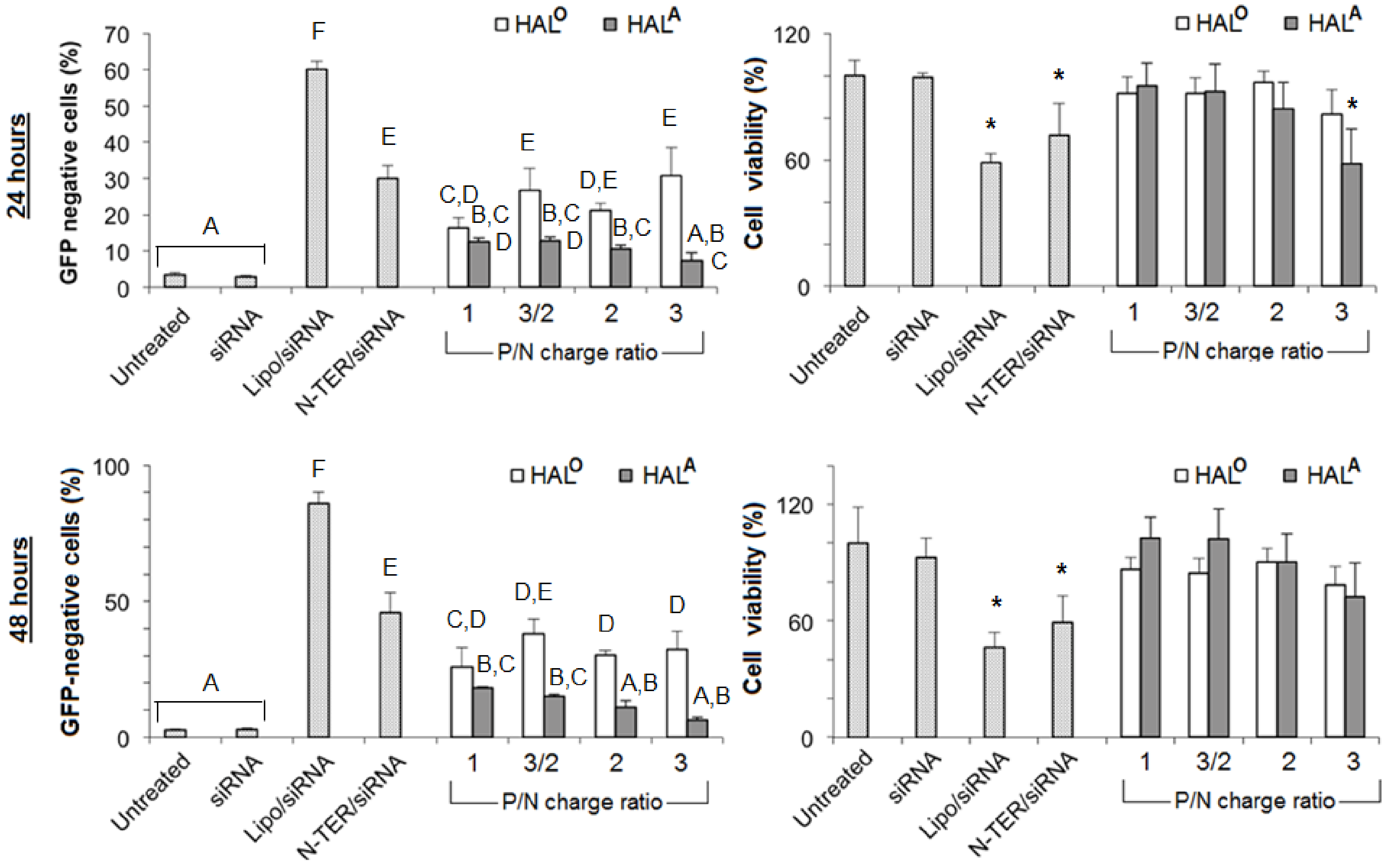

Figure S7. Peptide mediated RNAi. (Left) GFP silencing measured by flow cytometry in Flp-In TREx-293 cells following incubations with peptide/siRNA complexes at different charge ratios. The silencing is expressed in percentage with the total cell counts of an untreated GFP-positive population taken as 0%, allowing for false positive samples. (Right) Metabolic activity (cell viability) of the cells measured by alamarBlue® metabolic activity assay and expressed as a function of total absorbance and expressed in percentage (untreated cells taken as 100%), following incubations with peptide/siRNA complexes at different charge ratios. Error bars represent the standard deviation of at least three replicates. Results were analysed using one-way ANOVA followed by Tukey's test for multiple comparisons ($p < 0.05$). Different letters represent groups with statistically different means (*left*). (*) denotes groups with a mean statistically different from the control conditions (*right*).

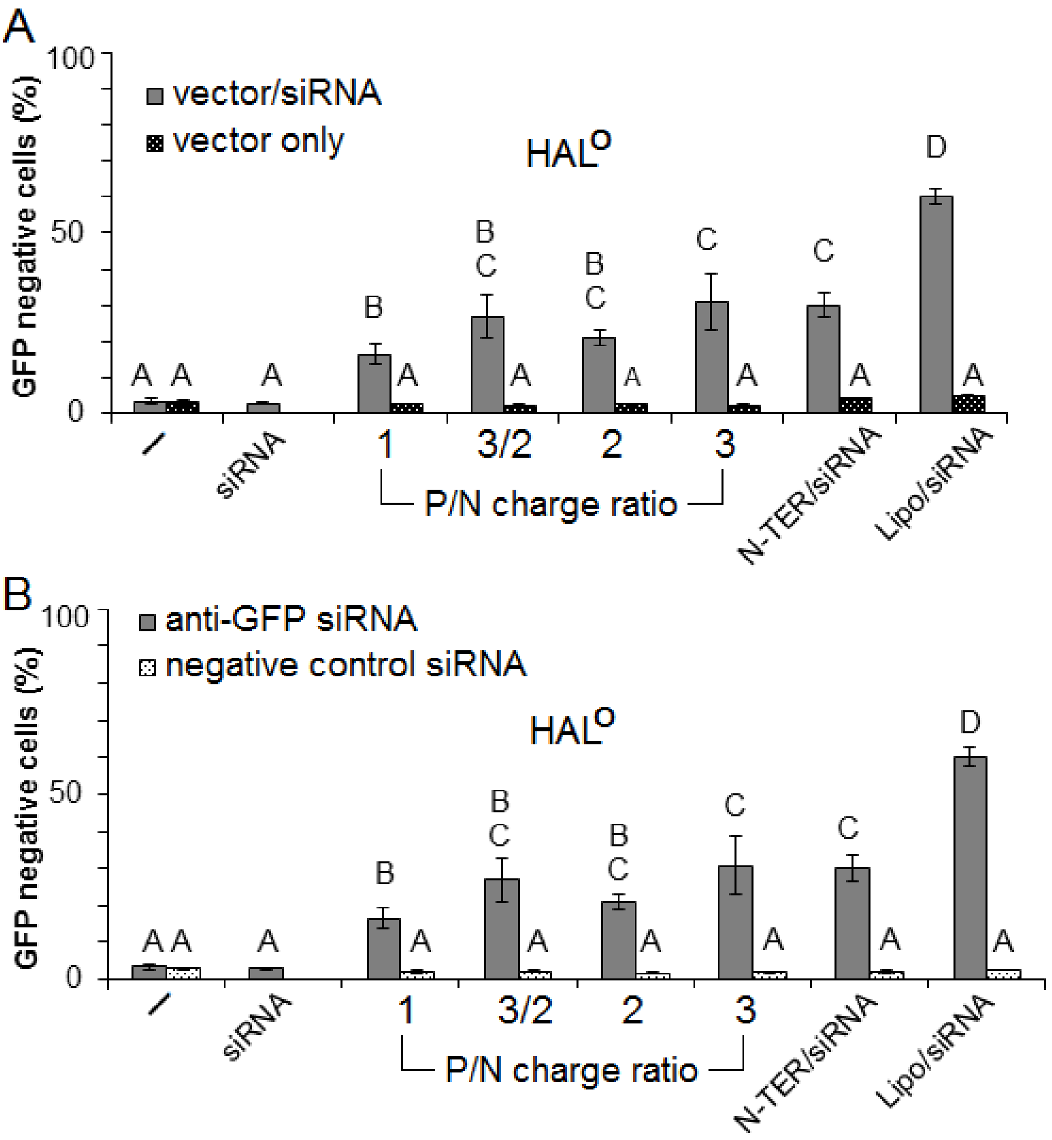

Figure S8. RNAi. GFP gene silencing after 24-h incubation at 37°C mediated by (A) vectors alone (negative controls) and in complex with anti-GFP siRNA, and (B) vectors in complex with anti-GFP siRNA and non-specific siRNA (negative control). The silencing is expressed in percentage with the total cell counts of an untreated GFP-negative population taken as 100%. Error bars represent the standard deviation of three replicates. Results were analysed using one-way ANOVA. Different letters represent groups with statistically different means ($p < 0.05$).

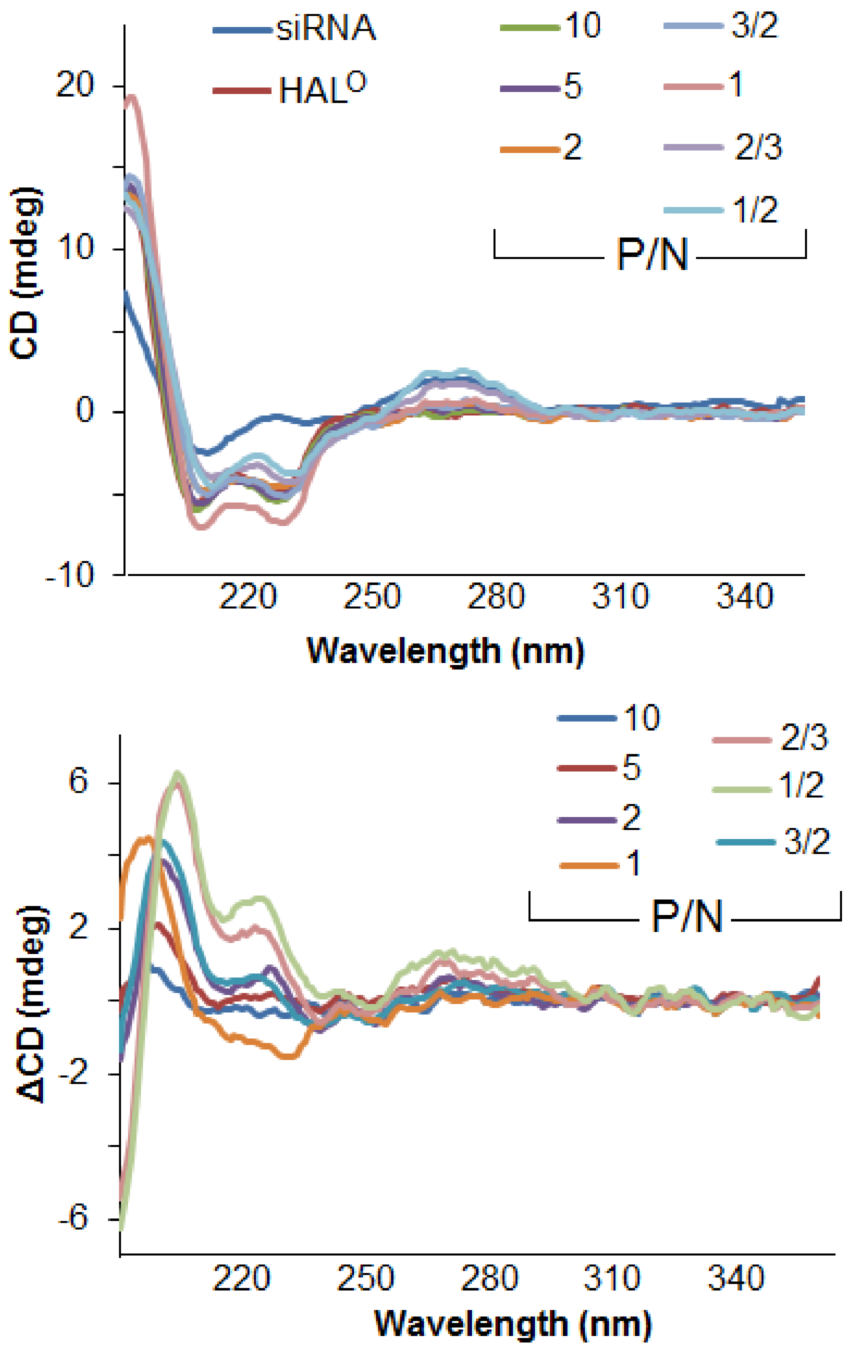

Figure S9. HAL^{O}/siRNA complexes at different P/N charge ratios. (Upper) Raw SRCD spectra and (lower) deferential CD spectra after subtracting the individual spectra for HAL^{O} and siRNA.

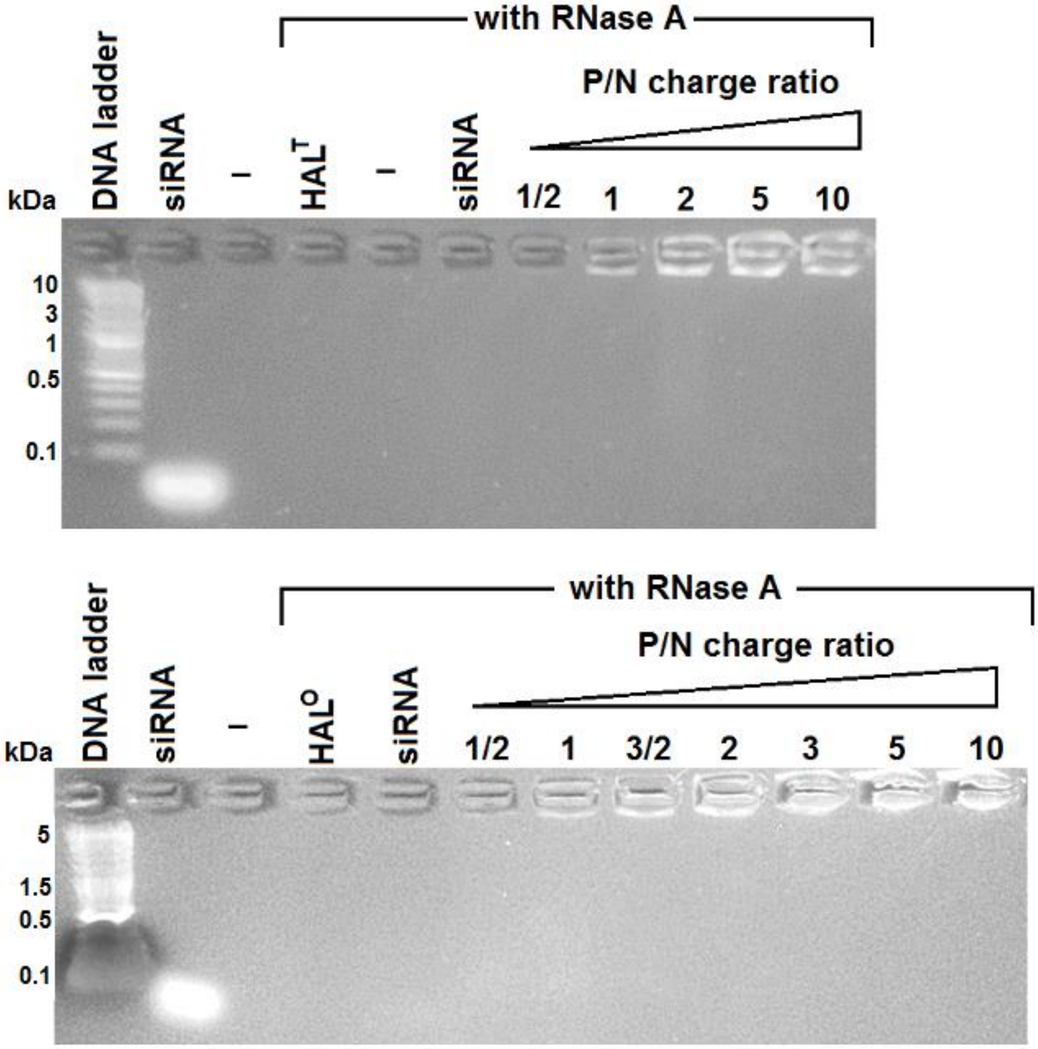

Figure S10. Agarose gel electrophoresis of HAL^T/siRNA (upper) and HAL^O/siRNA (lower) complexes at different P/N charge ratios in the presence of RNase A. Note: intact, peptide-protected siRNA is retained in the wells (increasing fluorescence) with increasing P/N charge ratio.

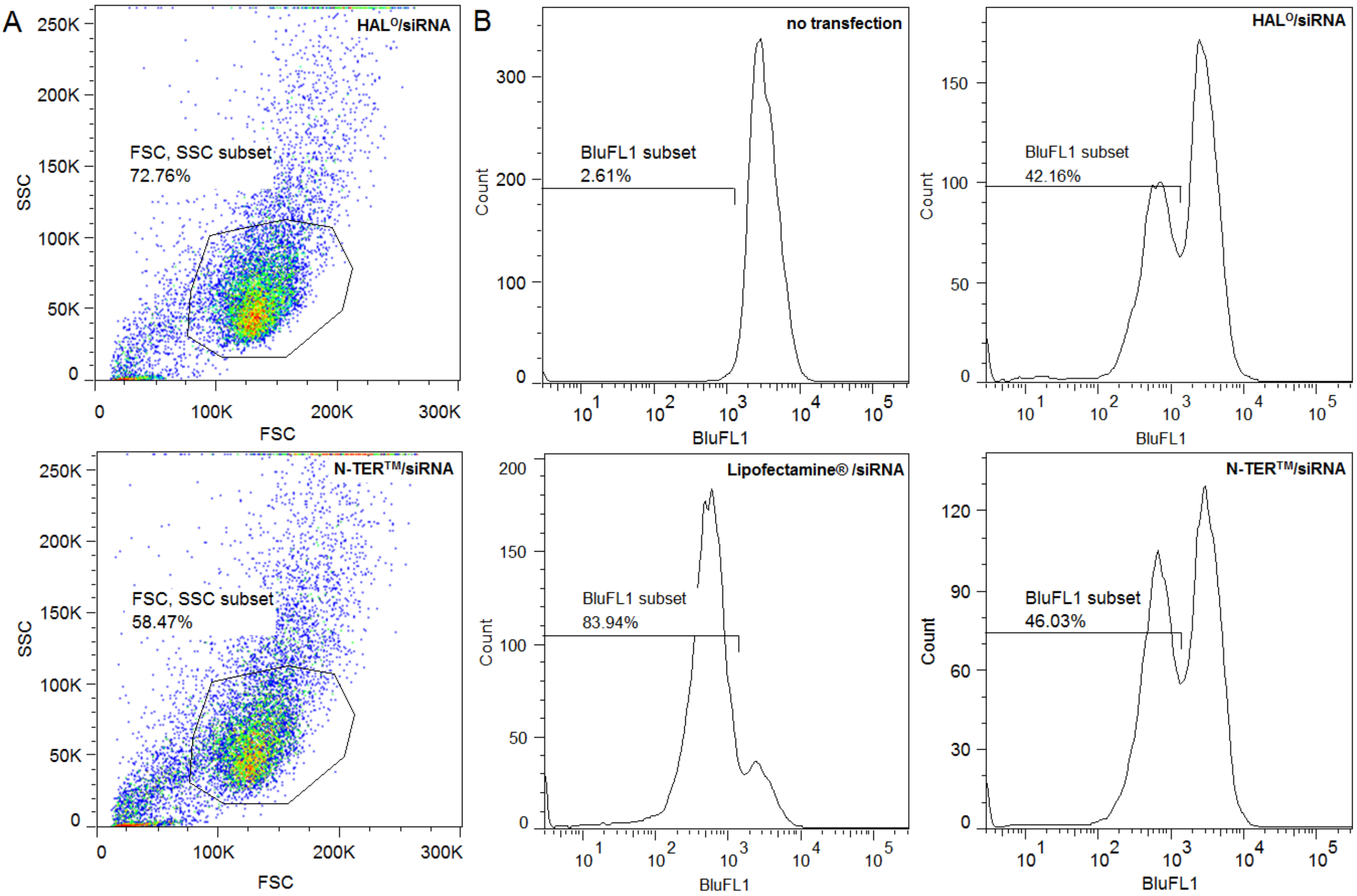

Figure S11. Flow cytometry charts used to measure GFP knockdown in Flp-In T-REx-293 cells stably expressing GFP after 48-h treatments with siRNA complexed with controls (Lipofectamine® and N-TER^{TM}) and HAL^{O} (at 3/2 P/N ratio). (A) Cell population gating: at least 10^4 events were gated (circled in the charts) from each $\geq 10^5$ subset measured for each sample by forward scatter and side scatter (X and Y axis, respectively) on the 488-nm laser (B) to extract and analyse 6-9 x 10^3 single viable cells. From the untreated cells, incubated without siRNA or transfection reagents, a GFP negative population was taken as 0% negative allowing for false positive samples. The gate was used on all other samples to express the relative population of GFP-negative cells in percentage.
